# Diabetes and Cognitive Impairment: A Role for Glucotoxicity and Dopaminergic Dysfunction

**DOI:** 10.3390/ijms222212366

**Published:** 2021-11-16

**Authors:** Francesca Chiara Pignalosa, Antonella Desiderio, Paola Mirra, Cecilia Nigro, Giuseppe Perruolo, Luca Ulianich, Pietro Formisano, Francesco Beguinot, Claudia Miele, Raffaele Napoli, Francesca Fiory

**Affiliations:** 1 Department of Translational Medical Sciences, University of Naples Federico II, 80131 Naples, Italy; francescachiara.pignalosa@gmail.com (F.C.P.); antonella.desid@gmail.com (A.D.); paolamirra06@gmail.com (P.M.); c.nigro@ieos.cnr.it (C.N.); giuseppe.perruolo@unina.it (G.P.); lulianic@unina.it (L.U.); fpietro@unina.it (P.F.); beguino@unina.it (F.B.); napoli@unina.it (R.N.); francesca.fiory@unina.it (F.F.); 2 URT “Genomic of Diabetes”, Institute of Experimental Endocrinology and Oncology, National Research Council, 80131 Naples, Italy

**Keywords:** diabetes mellitus, dopamine, cognitive impairment, glucotoxicity

## Abstract

Diabetes mellitus (DM) is a chronic metabolic disorder characterized by hyperglycemia, responsible for the onset of several long-term complications. Recent evidence suggests that cognitive dysfunction represents an emerging complication of DM, but the underlying molecular mechanisms are still obscure. Dopamine (DA), a neurotransmitter essentially known for its relevance in the regulation of behavior and movement, modulates cognitive function, too. Interestingly, alterations of the dopaminergic system have been observed in DM. This review aims to offer a comprehensive overview of the most relevant experimental results assessing DA’s role in cognitive function, highlighting the presence of dopaminergic dysfunction in DM and supporting a role for glucotoxicity in DM-associated dopaminergic dysfunction and cognitive impairment. Several studies confirm a role for DA in cognition both in animal models and in humans. Similarly, significant alterations of the dopaminergic system have been observed in animal models of experimental diabetes and in diabetic patients, too. Evidence is accumulating that advanced glycation end products (AGEs) and their precursor methylglyoxal (MGO) are associated with cognitive impairment and alterations of the dopaminergic system. Further research is needed to clarify the molecular mechanisms linking DM-associated dopaminergic dysfunction and cognitive impairment and to assess the deleterious impact of glucotoxicity.

## 1. Introduction

Diabetes mellitus (DM) is a heterogeneous chronic metabolic disorder, characterized by hyperglycemia, representing a global epidemic public health problem [1]. Premature morbidity and mortality of DM are due to long-term diabetic complications [2], including retinopathy, nephropathy, peripheral vascular disease, and heart disease (micro- and macro-vascular disease) [3]. Hyperglycemia brings together type 1 and type 2 diabetes, the two most common forms of DM, which differ in both epidemiology and etiology. Type 1 diabetes (T1D) is mostly a juvenile-onset disease due to autoimmune destruction of pancreatic beta cells, leading to an absolute deficiency in insulin production. Type 2 diabetes (T2D) includes 90–95% of diabetes cases and is a pathology typical of the elderly, resulting from insulin resistance accompanied by progressive beta cells deficit [4]. Interestingly, since 1922 [5], the idea that both T1D [6] and T2D [7] are accompanied by a worsening of cognitive function has emerged. Epidemiological studies showed that diabetes is associated with an increased risk of dementia [8] and less serious cognitive dysfunctions [9]. The severity of cognitive deficit depends on diabetes type, age of onset, and co-occurrence of complications and comorbidities [10]. Magnetic resonance imaging evidenced that diabetes is associated with structural changes in the brain. In particular, T1D patients feature frontal gray matter atrophy [11] and disturbed brain networks [12]. On the other hand, in T2D patients, gray matter loss is present in the prefrontal, hippocampus, amygdala, insular, cingulate, cerebellum, caudate, basal forebrain, and thalamus areas [13] and white matter loss is evident in frontal and temporal regions. In addition, magnetic resonance imaging also revealed more frequent cerebral infarcts in T2D subjects [14]. On a functional level, T1D patients feature slowing of mental speed and flexibility, information processing, and psychomotor and visuospatial functions [15,16] early in the disease [17]. Several studies indicated that the entity of T1D-associated cognitive dysfunction relies on age at diagnosis. Worse neuropsychological performances have usually been observed in T1D diabetic children diagnosed before the age of 7 [18]. In more detail, two different phenotypes depending on age onset have been recognized according to pediatric studies. Indeed, T1D patients with early onset, between 4 and 6 years old, feature potential clinically significant impairments in all cognitive functions, including learning and memory. In contrast, T1D diagnosed after the age of 6 or 7 is associated only with alterations in verbal intelligence and psychomotor speed and sometimes in executive functions but without changes in learning and memory [18,19]. When a large sample of T1D subjects was followed for 18 years, moderate long-term declines in cognitive function were observed [3]. Interestingly the development of microvascular complications, such as retinopathy and neuropathy, is accompanied by a faster cognitive decline over time and by worse cognitive performances in adults affected by T1D [20]. T2D is also associated with an increased risk for cognitive impairment and dementia [21]. Cognitive dysfunction has been observed not only in old T2D patients (age of 50–70), when cognition assessment was assessed by MMSE (Mini Mental State Examination) and 3MS (Modified Mini-Mental State Examination) [22] but also in adolescents affected by T2D [23]. Moreover, cognitive performance gets worse with diabetes duration and is affected by age at onset. Indeed, poorer cognitive performance was observed in T2D patients with midlife onset (40–64 age). In contrast, “late life” onset (after 65 age) is not associated with cognitive impairment [24]. The execution of a comprehensive multidimensional spectrum of cognitive neuropsychological tests [25] allowed the clarification that people with T2D feature significant impairments in the domains of visual and verbal memory, attention and concentration, processing speed, executive function, and motor control [26]. Similarly to T1D, cognitive impairment is often associated with diabetic complications in T2D. Interestingly, a study performed in a population of 1046 T2D patients (age 60–75) revealed that, in men, worse cognitive function was associated with increased severity of diabetic retinopathy, suggesting that cerebral microvascular disease could be involved in the cognitive decline observed in diabetes [27]. Permulter and coworkers [7] showed that cognitive decline in T2D individuals is associated with the degree of peripheral neuropathy, too. To confirm this, persistent albuminuria is associated with accelerated cognitive decline [28]. Nowadays, cognitive dysfunction can be considered a well-established complication of DM [29]. Different factors are involved in its pathogenesis, including diabetic macro and microangiopathy, cerebral vascular injury, amyloid and tau accumulation, poor glycemic control, and neurodegeneration, due to oxidative insult and mitochondrial dysfunction [30]. However, among DM complications, cognitive deficit remains the less addressed. Indeed, the underlying molecular mechanisms are far from being fully clarified and the research in this field is still ongoing. An interesting promising topic seems to be the potential role of alterations of the dopaminergic system in DM-associated cognitive dysfunction. In this review, we outline experimental evidence of the role of dopamine (DA) in the regulation of cognition and then we lay out the anomalies of the dopaminergic system observed in DM. Finally, we speculate about the potential impact of glucotoxicity on DM-associated dopaminergic dysfunction and cognitive deficit.

## 2. Dopamine Synthesis and Signaling

**Figure 1:**
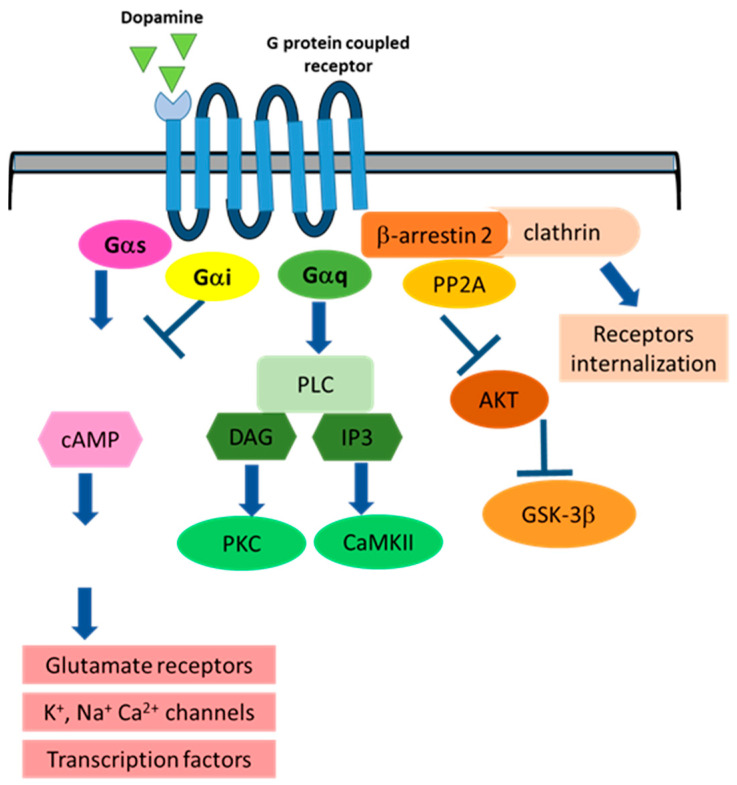
The main signaling pathways modulated by DA upon binding to specific G protein-coupled receptors.

DA is a neurotransmitter mainly synthesized in a two-step pathway in the cytosol of dopaminergic neurons, where the rate-limiting enzyme tyrosine hydroxylase (TH) hydroxylates L-tyrosine at the phenol ring, generating levodopa (L-DOPA). Then, DOPA decarboxylase (DDC) decarboxylates L-DOPA to DA [31]. The vesicular monoamine transporter 2 (VMAT2) imports DA into the synaptic vesicles, exocyted in response to changes of the membrane potential of the presynaptic terminal [32]. Once in the synaptic cleft, DA binds to regulatory presynaptic autoreceptors or to postsynaptic receptors [33,34,35,36], evoking an action potential. Dopaminergic signaling is stopped [37] through DA’s quick unbinding from receptors and consequent removal through reuptake in presynaptic neurons mediated by DAT (DA transporter) [38] or import by glial cells [39]. DA is then degraded through different catabolic pathways involving several enzymes, such as catechol-O-methyltransferase (COMT) [40], monoamine oxidase (MAO), and aldehyde dehydrogenase (ALDH), acting in sequence. The endproduct is homovanillic acid (HVA), a compound lacking known biological activity [41]. The details of DA signaling pathways have been extensively reviewed elsewhere [42]. Briefly, DA binds to different 7-transmembrane domain receptors divided in two major groups: D-1 like receptors, including D1 and D5 receptors, and D2-like receptors, including D2, D3, and D4. DA receptors are coupled to guanosine triphosphate-binding proteins (G proteins), able to modulate second messenger levels and, in turn, specific signaling pathways [43]. D1 and D5 receptors are localized in postsynaptic neurons, are coupled to stimulatory G protein Gαs, and activate adenylyl cyclase, leading to cAMP production and PKA activation. In contrast, D2 and D3, expressed both post- and presynaptically [44,45], and D4, widely expressed in the retina [46], are coupled to inhibitory G protein Gαi, which blocks the production of intracellular cAMP and PKA activity [43]. PKA phosphorylates several different substrates, such as the two major subtypes of glutamate receptors (α-amino-3-hydroxy-5-methyl-4-isoxazolepropionic acid receptor and N-Methyl-D-aspartate receptor), potassium, sodium [47], and calcium channels and specific transcription factors including CREB [48]. DA receptors are also able to induce the activation of phospholipase C (PLC) [49], leading to the activation of protein kinase (PKC) and CaMKII [50,51]. Beta arrestin 2 is involved in DA receptors’ signaling and regulation, too. Indeed, its binding to phosphorylated D2 receptors leads to the formation of a complex including the serine threonine kinase Akt and the phosphatase PP2A, resulting in constitutive activation of Akt substrates GSK3 alpha and beta [52]. Moreover, its binding to DA receptors induces receptors’ internalization and downregulation [53,54] (Figure 1).

## 3. Dopamine and Cognition

DA is historically known for its key role in the regulation of behavior and movement. Indeed, in 1973, for the first time, the strong undeniable association between striatal DA depletion and motor deficits characteristic of Parkinson’s disease (PD) emerged [55]. Later, signs of abnormal dopaminergic function were found in several diseases, such as schizophrenia [56] and attention deficit hyperactivity disorder [57]. The idea that DA could play a key role in cognitive function arises from the observation that these pathologies, due to anomalies of the dopaminergic system, are also characterized by cognitive impairment [58,59,60]. In particular, PD is characterized by alterations of several cognitive domains, including executive functions [61], attention [62], verbal fluency, visuospatial skill [63], episodic memory [64], and reasoning [65]. Interestingly, some of these cognitive domains are altered in DM, too [66,67,68,69]. Imaging studies performed in PD patients revealed a positive correlation between decreased DA levels and cognitive impairment [70,71,72,73]. Interestingly, aging-associated cognitive decline is also accompanied by various modifications of the dopaminergic system [74]. The observed changes concern a reduction of DA receptor and transporter density [75,76,77,78]. In more detail, in healthy subjects, molecular imaging studies highlighted an age-related decrease in striatal dopamine transporter (DAT) density, paralleled by a worse performance on several tasks, including episodic memory, executive functioning, and verbal learning tasks [79,80].

Similarly, healthy aging is also characterized by a loss of D2 receptors in both striatal and extrastriatal areas and by simultaneous cognitive deficits [81,82,83]. An increase of DA catabolism [84] and alterations of DA synthesis [85] were observed too. Thus, dopaminergic dysfunction could mediate the association between aging and cognitive decline [86]. Moreover, impaired DA transmission was observed in diseases featuring cognitive deficit, such as Alzheimer’s disease [87], autism [88], and Huntington’s chorea [89]. Several in vivo data obtained in different experimental models undoubtedly link DA release to cognitive function. Experiments of in vivo microdialysis allowed evidence of increased DA release in the prefrontal cortex (PFC) of rats [90] and monkeys [91] during working memory tasks. Accordingly, the application of DA modulates neuronal “memory field” activity of PFC neurons [92]. DA relevance for cognition was then confirmed by both experimental manipulation of the dopaminergic system in animal models and by pharmacological studies in humans. Several studies that aimed to clarify DA involvement in cognitive function were performed in rodents. Simon and coworkers showed that in rats, the bilateral injection of 6-hydroxydopamine (6-OHDA) into the lateral septum selectively abolishes dopaminergic innervation and leads to deficits in spatial-memory tasks, without significantly damaging endogenous noradrenergic and cholinergic systems [93,94]. Similarly, in rhesus monkeys, depletion of DA in PFC severely impairs working memory [95]. The entity of the deficit is comparable to that observed when the PFC itself is ablated [96,97]. Interestingly, no alterations of working memory were observed in monkeys subjected to the depletion of other neurotransmitters [98]. Moreover, the deleterious effect of depletion of DA in PFC was reverted by treatment with DA receptor agonists, supporting the selective relevance of DA for working memory [95,99]. This finding was further confirmed by results obtained in rhesus monkeys when selective antagonists of the D1 dopamine receptor were locally injected into the PFC and altered mnemonic processes [100]. Experiments with agonists and antagonists of the D1 dopamine receptor further strengthen the idea that cognitive processing strongly depends on an optimal level of DA. Indeed, both excessive and inadequate activation of D1 receptor impairs working memory ability in both monkeys and rodents [101,102,103]. Analogous studies, performed in humans, revealed that the treatment of healthy human subjects with the selective D2R agonist bromocriptine facilitates spatial working memory [104]. Similarly, the administration of pergolide, an agonist for both D1 and D2 family receptors, improved performances in working memory tasks [105]. More detailed dose–response experiments with dopaminergic drugs supported the hypothesis that the complex functional relationship between DA and working memory is regulated by a nonlinear inverted U-shaped dose–response curve [106], where both low and excessive doses of DA impair working memory performance [98]. This trend is likely influenced by baseline levels of DA [107] and may depend on the differential effects of DA receptor activation in the striatum and PFC [108]. More recently, effects of D1 and D2 receptors agonists have been better investigated across multiple tasks exploring several cognitive domains, such as memory, flexibility, and learning in non-human primates, unveiling dose- and task-specific actions and strongly suggesting distinct cognitive functions of DA receptors in the PFC and striatum [106,108]. Indeed, cognitive control deficit is often due not only to malfunctioning of PFC, but also to impaired striatal DA transmission. Studies performed with 1-Methyl-4-phenyl-1,2,3,6-tetrahydropyridine (MPTP) in both animal and human models disclosed the relevance of dopaminergic signaling in the striatum for cognitive functions. Stern Y and coworkers in 1990 investigated general intellectual function, construction, language, memory, executive function, attention, and reaction time in MPTP-exposed individuals, characterized by reduced uptake of labeled 6-fluorodopa into the striatum. They featured significantly worse performances in a specific set of cognitive functions mediated by the dopaminergic system [109]. Similarly, in rats, intranigral administration of MPTP causes a partial lesion in the substantia nigra, compact part (SNc), and a specific loss of DA in the striatum, inducing habit learning and working memory deficits [110,111]. Memory acquisition and retention processes were impaired, too [112]. The idea that an impaired nigrostriatal system participates in cognitive dysfunction was further supported by bilateral lesion obtained by 6-OHDA injection into the ventrolateral neostriatum, leading to altered working memory, accompanied by striatal DA depletion [113]. Interestingly, intrastriatal administration of D2 receptor agonist to rats leads to improved cognitive performance [114,115]. Similar results were obtained in MPTP-lesioned monkeys upon systemic injection of D1 receptor agonist [116], suggesting that mnemonic processes need normal stimulation of striatal DA receptors. Studies performed in PD patients highlighted that DAT availability in the caudate, anterior putamen, and ventral striatum was also directly associated with attention/working memory, frontal/executive, and visuospatial functions [117]. Some experimental evidence supports a role for DA in the regulation of attentional function [118], too. In rats, bilateral 6-OHDA lesions of the terminal area of the nigrostriatal DA system induce a partial DA denervation of the striatum, leading to attentional deficits [119,120]. DA’s role in the modulation of attentional processes was further confirmed by the observation that the stimulant drug methylphenidate improves attention in ADHD patients by enhancing DA signaling in the ventral striatum [121]. Interestingly, in intrastriatal bilateral 6-OHDA rats, the altered attentional performance on a reaction time task was significantly improved by co-administration of L-DOPA with piribedil, an agonist of D2/D3 receptors [122]. Several pharmacological studies then allowed better clarification of the role of DA receptors in attentional control, showing that the D2 receptor seems to be more involved in attentional control than the D1 receptor [123,124]. More recent findings in mice revealed that DA released in the dorsal hippocampus from the locus coeruleus has a role in increasing selective attention to relevant features of the environment, promoting spatial learning via D1/D5 receptor [125]. Moreover, attentional performances in healthy individuals are affected by genetic factors, such as SNPs in dopaminergic genes. Several studies suggest that dopaminergic polymorphisms influence selective aspects of cognition [126,127]. For instance, COMT val158met polymorphism modulates dopaminergic signaling affecting the function of the cingulate cortex during attentional control [128]. In addition, the allelic mRNA expression analysis of human brain autopsy tissues, followed by the SNP scanning of the DRD2 locus, allowed the discovery of regulatory polymorphisms modulating DRD2 splicing, working memory network, and cognitive performance in healthy humans [129]. Similarly, polymorphisms in several other dopaminergic genes, such as DRD1 and DRD4, modulate the executive function and working memory processes in healthy subjects [130]. Finally, dopaminergic system integrity is also necessary for several other cognitive functions, such as intertemporal choice [131], task-switching [132], response inhibition [133], and arousal [134].

## 4. Alterations of Monoamines System in Diabetes

An undeniable cross talk between the brain monoamine neurotransmitter system and glucose homeostasis has been widely described in the literature. Both T1DM and T2DM are indeed associated with deleterious changes in the brain monoaminergic system that play a role in the pathogenesis of DM and of DM-associated neurodegeneration [135,136,137]. In particular, several research groups described alterations in the metabolism and content of adrenaline, norepinephrine, serotonin, and DA in different specific brain areas of diabetic rodents and humans [138,139,140,141,142]. However, DA and serotonin appear to be the main regulators of cognitive function [143]. 

### 4.1. Serotonergic System 

Studies performed in different experimental models evidenced alterations of the serotonergic system in DM. Indeed, in the CNS of T2DM patients, several perturbations of the serotonergic system were observed, such as a decreased free tryptophan amount, impaired serotonin biosynthesis, and alterations of serotonin receptors [144,145,146]. Changes of the serotonin content in the medial and lateral hypothalamus were evidenced in diabetic patients [139] as well as in specific brain regions of STZ- and alloxan-treated rats [138,147]. In more detail, in STZ-induced T1DM rodent models, in vivo intracerebral microdialysis studies under free-moving conditions evidenced a significant decrease of serotonin levels in the hypothalamus, hippocampus, brainstem, cortex, and ventromedial hypothalamus [148,149,150,151,152]. Different molecular mechanisms have been proposed to explain a reduction of the serotonin amount in STZ models, including increased activity of MAO-A/MAO-B, higher serotonin reuptake by serotonin transporter SERT, and elevated plasma levels of branched-chain amino acids, which compete with tryptophan to cross the blood brain barrier [153]. Similarly, a murine model of T2DM induced by a prolonged high-fat diet features a reduction of serotonin extracellular levels in hippocampal and a hypersensitization of inhibitory 5-hydroxytriptamine A1 serotonin autoreceptors in dorsal raphe nuclei, leading to an inhibition of the serotonergic circuit. These deleterious changes contribute to T2DM-associated mood and eating disorder [154] and lead to lower insulin sensitivity and metabolic dysfunctions, playing a role in T2DM exordium [135]. Indeed, serotonin and serotonergic drugs ameliorate peripheral glucose uptake, glucose tolerance, and insulin sensitivity in diabetic rat models and in diabetic patients [155,156,157,158]. Moreover, a critical role of serotonin in cognitive processes was pointed out by experimental manipulation of tryptophan levels in murine models, primates, and humans [159,160,161,162,163] and by the finding that anomalies of serotonin content and signaling are involved in cognitive decline associated with Alzheimer’s disease and ageing [164,165]. Reduced serotonin transmission leads to impaired learning and memory function, while augmented serotonin transmission ameliorates cognitive performance both in rodents and humans [166]. Pharmacological strategies that aim to restore serotonin levels have beneficial effects both in diabetic patients and in T2DM animal models, improving cognitive function and metabolic parameters [144,145,146]. A key role for serotonergic alterations in cognitive dysfunction also emerged in rats in the later stage of T1DM, characterized by reduced spatial memory and learning ability [167]. An interesting topic is the intricate cross talk between serotonergic and dopaminergic systems, clearly highlighted by neurological and pharmacological studies and by evidence obtained in knockout murine models [168,169,170,171]. The serotonergic system modulates dopaminergic transmission by 5-HT2A receptors and there is also evidence of the existence of heteromers 5-HT2A/D2 receptors expressed in dopaminergic cells of different brain areas [172,173]. A further level of serotonergic and dopaminergic interaction occurs at the postsynaptic level within the PSD (post synaptic density), involving several scaffolding protein and signaling molecules [174]. The interplay between dopamine and serotonin systems obviously has both physiological and pathological implications and research is actually focused on its relevance for antipsychotic action and for the discovery of innovative therapeutic target for psychosis [175]. At variance, its contribution to DM-associated cognitive dysfunction has not been investigated yet.

### 4.2. Dopaminergic System

Some experimental data highlighted an interplay between glucose metabolism and the dopaminergic system. Indeed, it has been shown that, both in rodents and humans, modulation of striatal and systemic DA levels impinges on whole body glucose metabolism [176] and energy homeostasis [177,178]. In murine models, optogenetic activation of nucleus accumbens (NAc) cells expressing DRD1 improved glucose tolerance and insulin sensitivity [176]. In addition, DRD2 is implicated in the modulation of insulin secretion [179] and different authors showed that systemic treatment with bromocriptine, a DRD2 agonist, improves insulin sensitivity [180] and glucose tolerance in humans [181]. Similar results were observed in obese hamsters, too [182]. In contrast, systemic DA depletion leads to a decrease of striatal DA levels and in turn to a reduction of insulin sensitivity in healthy subjects [176], as well as antipsychotics, inhibiting DA receptors, and inducing hyperinsulinemia and glucose intolerance [183,184]. Moreover, striatal DA receptors regulate the expression of insulin receptor and of the neuron-specific glucose transporter GLUT-3 in streptozotocin diabetic rats [185].

On the other hand, the presence of DM promotes neurodegeneration and impairs dopaminergic neurotransmission [186]. This is consistent with the finding that the two major players of DM, such as hyperglycemia and relative insulin deficiency, can alter the dopaminergic system. Indeed, insulin is a key regulator of both neurons’ survival and DA metabolism. First of all, insulin protects rat hippocampal cells in culture by oxygen-glucose deprivation [187] and has neuroprotective action against H2O2 in retinoic acid (RA)-differentiated SH-SY5Y cells [188]. Similarly, in rats, insulin protects dopaminergic neurons of substantia nigra against 6-OHDA toxicity [189]. Importantly, impaired insulin signaling alters DA homeostasis [190,191,192,193] and the ablation of insulin receptors in dopaminergic neurons interferes with DA action on control of food intake [194]. Accordingly, it was recently shown that in ex vivo differentiated human dopaminergic neurons and in SH-SY5Y cells in culture, insulin resistance is accompanied by mitochondrial dysfunction, increased ROS levels, and increased expression of alpha-synuclein [195]. Insulin is a known modulator of DA synthesis and turnover, too. As proof of this, NIRKO mice, carrying a brain-specific knockout of the insulin receptor, feature increased DA turnover in the striatum and NAc, resulting in decreased DA signaling [196]. Moreover, in non-diabetic rats, insulin injection increases DA levels in NAc [197]. Interestingly, insulin is able also to regulate the expression of TH [198] and increase DA uptake by DAT [190,199,200]. Similarly, opportune glucose intake to the brain is crucial for both dopaminergic neurons homeostasis and DA metabolism. Studies focusing attention on the hyperglycemia effect in dopaminergic neurons revealed that they are prompted to apoptosis by chronic glucose exposure through oxidative damage [201,202,203]. In PC12 cells, chronic incubation with high glucose augmented depolarization-induced DA release [204], and in healthy human subjects, blood glucose levels are related to cerebrospinal fluid concentrations of the DA metabolite homovanillic acid [205]. In rats, variations of ambient glucose levels in substantia nigra, obtained by use of microdialysis probes, produce different effects on DA release, depending on both the concentration and duration of infusion. Glucose action seems to also involve ATP-sensitive K+ channels and regulate the efflux of other neurotransmitters, too. However, in the nigrostriatal pathway, glucose infusion seems to increase DA release when glucose availability is low while decreasing DA release when glucose is abundant [206]. Interestingly, the huge impact of glucose and insulin on the dopaminergic system has recently been observed in *Caernorhabditis elegans*, too [207]. Thus, given the key role of insulin and glucose in DA homeostasis, it is not surprising that dopaminergic function is altered in DM. Studies evidencing DM-associated dopaminergic dysfunction were performed in DM animal models for the vast majority. At variance, few studies about dopaminergic dysfunction have been conducted in diabetic patients, thus it is not clear yet if there are substantial differences in dopaminergic alterations between T1DM and T2DM patients. Some authors described an increase of DA levels during DM in specific brain regions of alloxan- or streptozotocin (STZ) rats [138,208], as well as diabetic patients [139]. The selectivity of DA content alterations was further confirmed by Ezzeldin et al. They found a reduced DA amount in the cerebral cortex, midbrain, and brainstem regions but augmented in the cerebellum and thalamus/hypothalamus [140]. However, in later years, there are more detailed studies supporting a reduction in DA levels in different brain areas during DM. In particular, in the hippocampus of STZ rats and spontaneously diabetic WBN/Kob rats (WBN rat), a reduction of DA levels and release was observed [151]. Interestingly, the reduced DA content in the hippocampus of STZ diabetic rats is paralleled by compensatory upregulation of DRD1 and DRD2 expression and contributes to a cognitive deficit [209]. Gallego et al. observed a selective reduction of DA content in the dopaminergic nigrostriatal system in STZ rats, also highlighting that the alterations of catecholamine metabolism depend on the severity and duration of DM [210]. Very recently, dopaminergic alterations induced by long-term hyperglycemia were investigated in detail in STZ rats. The glucose amount was increased in the midbrain and striatum, but preferential neurodegeneration of the nigrostriatal pathway, accompanied by astrogliosis and loss of microglial cells, was observed with aging. The higher vulnerability of the nigrostriatal pathway to long-term hyperglycemia probably results from an elevated basal oxidative burden paralleled by low levels of antioxidant defense [211]. Similar results were obtained by Pérez-Taboada et al., who found decreased levels of DA and related metabolites in the striatum of both STZ-treated mice and diabetic db/db mice. A specific reduction of the expression of protein regulating DA neurotransmission and stimulus-dependent striatal DA release, such as DAT, VMAT2, and Girk2, was observed too [186]. It is worthy of notice that the expression of several proteins, involved in DA synthesis and degradation, including TH, MAO, COMT, and SNCA, is deregulated in DM. DM deleterious effects on TH function have been known since the 1980s, when modifications of the amount of aminoacids precursors and TH activity, leading to reduced striatal DA metabolism, were observed in STZ diabetic rats [212]. Several authors obtained similar results. Indeed, a progressive decrease in TH activity was observed in STZ-treated Sprague-Dawley rats by Bitar and coworkers [213]. Moreover, in STZ-treated rats, TH mRNA was increased in the locus coeruleus but decreased in the ventral tegmental area/substantia nigra pars compacta [214]. Similarly, reduced TH activity in terminal fields for noradrenergic and dopaminergic neurons was observed in experimental diabetes [150,151,215], while genetically diabetic Wistar rats feature decreased levels of immunoreactive TH [216,217], too. Interestingly, MAO shows significantly increased activity in diabetics’ platelets [218] and an augmented expression in NIRKO mice [196]. Finally, it has been shown that some functional polymorphisms in the COMT gene, responsible for the modulation of its enzymatic activity in PFC, are significantly associated with T2DM [219,220,221]. DM’s deleterious effect on the dopaminergic system was recently confirmed in human studies, too. Indeed, diabetic patients feature striatal dopaminergic deficits and elevated levels of proteins involved in neurodegeneration, such as tau and SNCA, in cerebrospinal fluid [222].

## 5. Glucotoxicity Role in Dopaminergic Dysfunction and Cognitive Impairment

Chronic hyperglycemia, typical of DM, seriously damages organs and tissues, leading to the onset of diabetic complications and giving rise to glucotoxicity. Among the involved mechanisms, overactivation of the hexosamine and polyol pathways, activation of protein kinase C (PKC), and increased intracellular formation of advanced glycation end products (AGEs) have been described [223]. The glucotoxicity condition is characterized by abnormal intracellular accumulation of reactive dicarbonyls, such as methylglyoxal (MGO), glyoxal, and 3-deoxyglucosone [224]. The α-ketoaldehydes MGO represents the most potent glycating agent, promoting the endogenous non-enzymatic glycoxidation of proteins, lipids, and nucleic acids and the consequent formation of advanced glycation end products (AGEs). In more detail, MGO targets arginine and lysine residues of proteins and deoxyguanosine in DNA, leading to the formation of AGEs and MGO-derived DNA adducts, respectively. MGO mainly comes from spontaneous degradation of triosephosphates deriving from glycolysis and, to a lesser extent, from the catabolism of threonine, catabolism of ketone bodies, degradation of glycated proteins, and lipid peroxidation. MGO and MGO-derived AGE plasma levels are higher both in T1DM and T2DM. Indeed, hyperglycemia increases glycolytic flux and/or decreases the activity of MGO detoxifying systems, promoting MGO accumulation [225]. The undeniable role of MGO and AGEs in DM and its vascular complications has been extensively reviewed elsewhere [226,227]. Briefly, it has been shown that MGO affects insulin secretion [228] and promotes insulin resistance in different tissues, such as skeletal muscle [229] and endothelium, both in vitro and in vivo [230]. Different mechanisms underlie MGO deleterious action on endothelial function, including the downregulation of specific miRNAs [231,232] and the increased accumulation of the antiangiogenic factor HoxA5 [233]. Several recent studies highlighted the relevance of MGO and AGEs not only in micro- and macrovascular DM-associated complications, but also in neurodegenerative diseases and in cognitive dysfunction [234,235,236,237]. A great deal of evidence in the literature demonstrates the deleterious effects of MGO in neuronal cells. Most of the studies have been performed in neuronal cells from the hippocampus, a brain region essential for cognitive processes. Upon MGO exposure, hippocampal neurons obtained from fetal hippocampi of Sprague-Dawley rats undergo apoptosis through both mitochondrial and Fas receptor-mediated pathways. This phenomenon is accompanied by an unbalance of the cytokines network and by a significant alteration of antioxidant capacity and detoxification mechanisms. In addition, other authors describe MGO-induced inhibition of catalase enzymatic activity and protein expression and an increase of NGF and proinflammatory cytokine IL-1beta levels in this cellular model. Similar results were obtained ex vivo in slices of the cerebral cortex and hippocampus from the neonatal rat brain, where MGO elicited its toxicity through both a ROS-dependent ERK1/2 pathway and ROS-independent p38 and JNK pathways [238]. Incontrovertible proof of the impact of glucotoxicity on DM-associated cognitive dysfunction in vivo comes from both animal and human studies. Huang and coworkers showed that in STZ diabetic rats, the increase of blood glucose levels correlates with increased serum MGO. High MGO levels increase the percentage of apoptosis in hippocampal neurons, altering the amount of cleaved caspase-3, Bcl-2, and Bax [239]. Subsequent animal studies further confirmed that neurotoxicity due to an increased amount of MGO may play a key role in DM-associated cognitive decline. Indeed, in Wistar rats, intracerebroventricular infusion of MGO impairs GLO1 (glyoxalase 1) activity, increases AGE content, and leads to cognitive deficit, altering the hippocampus but not the frontal cortex. In more detail, MGO injection impairs discriminatory memory without affecting learning-memory processes and locomotion behavior [240]. In addition, the novel object recognition task and Y-maze test showed that short- and long-term memory and short-term spatial memory are impaired by intracerebroventricular injection of MGO in rats [241]. Similarly, dietary AGEs can worsen learning and memory and induce mitochondrial dysfunction in mice [242]. Glucotoxicity relevance for neurodegeneration has been explored in human studies, too. First of all, a role for MGO and MGO-derived AGEs in neurodegenerative diseases, such as Alzheimer’s disease and Parkinson’s pathogenesis, has been evidenced [243,244]. In particular, protein glycation adduct levels are increased in CSF of Alzheimer’s disease patients and MGO levels are increased in the serum of individuals with mild cognitive impairment [245]. Importantly, in non-demented elderly subjects, higher serum MGO amount [246] and dietary AGEs [247] are associated with a faster cognitive decline and faster rate of decline in memory, respectively. Moreover, increased serum MGO levels are associated with poorer memory, worst executive function, and lower gray matter volume [248], supporting the idea that glucotoxicity is involved in cerebral atrophy and cognitive dysfunction. Interestingly, experimental data suggest that glucotoxicity and MGO in particular can impinge on the dopaminergic system, too. First, in frozen human brain tissue, a neurotoxin called ADTIQ (1-acetyl-6,7-dihydroxy-1,2,3,4-tetrahydro-isoquinoline) was identified, deriving from the reaction of methylglyoxal with dopamine and particularly abundant in the putamen and caudate nucleus regions of Parkinson’s patients [249]. Interestingly, ADTIQ has neurotoxic properties, and its levels are significantly increased in a cell model of hyperglycemia, diabetic rat brain [250], and transgenic mice expressing mutant forms of alpha-synuclein [251]. Interestingly, MGO treatment of N2A cells overexpressing α-syn induces in the cytoplasm the formation of α-syn aggregates positive for anti-CML antibody staining. Similarly, in mice, unilateral stereotaxic administration of MGO into the substantia nigra leads to the formation of α-syn aggregates accompanied by a significant reduction in protein levels of TH and of DJ-1, a protein with deglycase activity and that works as a sensor of oxidative stress [252]. Very recently, de Almeida and coworkers, performing the tail suspension test and Y maze spontaneous alternation test, discovered that MGO treatment induces depression-like behavior and impairs working memory in mice, inducing in parallel a significant reduction of dopamine and serotonin levels in the cerebral cortex [253]. Similar results were obtained by Szczepanik JC et al., who showed impairment, anxiolytic, and depressive-like behavior in Swiss mice memory. A daily administration of MGO for 11 days decreased dopamine levels and the Glo1 amount in the prefrontal cortex [254]. Finally, at the molecular level, it is known that MGO can regulate dopamine levels and the expression of dopaminergic genes, such as TH and DAT, in SH-SY5Y cells [255].

## 6. Conclusions and Perspectives

To date, cognitive decline undoubtedly represents a new emerging long-term complication of DM, leading to a lack of diabetes self-management and poor glycemic control. DM-associated cognitive deficit has a wide deleterious effect on life quality and significant consequences on the public healthcare system. However, its pathogenesis still remains obscure. In parallel, the relevance of the dopaminergic system for cognitive function has recently emerged, too. Hyperglycemia is known to be responsible for tissue damage leading to classical diabetic complications and impinges on dopaminergic neurons’ homeostasis, too. Indeed, hyperglycemia induces greater glucose permeation in the brain [256] and an increase of intracellular and extracellular glucose concentrations in the midbrain and striatum [212], leading to neuronal glucotoxicity through different mechanisms, such as mitochondrial dysfunction, oxidative stress, polyol pathway, hexosamine pathway, and accumulation of the glycating agent MGO, a precursor of AGEs [257]. In particular, recent evidence strongly suggests a key role for MGO in both DM-associated cognitive decline and dopaminergic dysfunction. Indeed, MGO impairs dopaminergic neurons’ survival and regulates dopamine levels in animal models. However, MGO’s deleterious effects on cognition and dopaminergic function probably involve several molecular mechanisms that still remain unexplored. For instance, the hypothesis that MGO could modify dopaminergic genes’ expression has not been deeply explored yet. It is noteworthy that epigenetic changes could potentially be relevant for MGO’s deleterious effect on cognitive and dopaminergic functions. However, this point has never been investigated. The elucidation of molecular mechanisms underlying MGO’s action is crucial for both the early identification of DM patients at risk of cognitive decline and the development of innovative therapeutic strategies for its treatment. Indeed, epigenetic modifications induced by MGO could represent novel potential biomarkers of the risk to develop dopaminergic dysfunction and cognitive impairment. Interestingly, epigenetic alterations can be induced or reverted by environmental factors, such as dietary factors. This feature provides an intriguing possibility to manipulate epigenetic mechanisms via dietary nutrients, thus improving both cognitive and dopaminergic dysfunction associated with DM.

## Data Availability

Not applicable.

## References

[1] World Health Organization (2019). ADA Classification of Diabetes Mellitus.

[2] Harding J.L., Pavkov M.E., Magliano D.J., Shaw J.E., Gregg E.W. (2019). Global trends in diabetes complications: A review of current evidence. Diabetologia.

[3] Reasner C.A. (2008). Reducing cardiovascular complications of type 2 diabetes by targeting multiple risk factors. J. Cardiovasc. Pharmacol..

[4] Petersmann A., Müller-Wieland D., Müller U.A., Landgraf R., Nauck M., Freckmann G., Heinemann L., Schleicher E. (2019). Definition, Classification and Diagnosis of Diabetes Mellitus. Exp. Clin. Endocrinol. Diabetes.

[5] Miles W.R., Root H.F. (1922). Psychologic tests applied to diabetic patients. Arch. Intern. Med..

[6] Bispham J.A., Hughes A.S., Driscoll K.A., McAuliffe-Fogarty A.H. (2020). Novel Challenges in Aging with Type 1 Diabetes. Curr. Diabetes Rep..

[7] Perlmuter L.C., Hakami M.K., Hodgson-Harrington C., Ginsberg J., Katz J., Singer D.E., Nathan D.M. (1984). Decreased cognitive function in aging non-insulin-dependent diabetic patients. Am. J. Med..

[8] Biessels G.J., Staekenborg S., Brunner E., Brayne C., Scheltens P. (2006). Risk of dementia in diabetes mellitus: A systematic review. Lancet Neurol..

[9] Koekkoek P.S., Kappelle L.J., van den Berg E., Rutten G.E., Biessels G.J. (2015). Cognitive function in patients with diabetes mellitus: Guidance for daily care. Lancet Neurol..

[10] Biessels G.J., Deary I.J., Ryan C.M. (2008). Cognition and diabetes: A lifespan perspective. Lancet Neurol..

[11] Hughes T.M., Ryan C.M., Aizenstein H.J., Nunley K., Gianaros P.J., Miller R., Costacou T., Strotmeyer E.S., Orchard T.J., Rosano C. (2013). Frontal gray matter atrophy in middle aged adults with type 1 diabetes is independent of cardiovascular risk factors and diabetes complications. J. Diabetes Complicat..

[12] Roy B., Ehlert L., Mullur R., Freeby M.J., Woo M.A., Kumar R., Choi S. (2020). Regional Brain Gray Matter Changes in Patients with Type 2 Diabetes Mellitus. Sci. Rep..

[13] Moran C., Phan T.G., Chen J., Blizzard L., Beare R., Venn A., Münch G., Wood A.G., Forbes J., Greenaway T.M. (2013). Brain Atrophy in Type 2 Diabetes. Diabetes Care.

[14] Brands A.M., Biessels G.J., de Haan E.H., Kappelle L.J., Kessels R.P. (2005). The effects of type 1 diabetes on cognitive performance: A meta-analysis. Diabetes Care.

[15] Shalimova A., Graff B., Gąsecki D., Wolf J., Sabisz A., Szurowska E., Jodzio K., Narkiewicz K. (2019). Cognitive Dysfunction in Type 1 Diabetes Mellitus. J. Clin. Endocrinol. Metab..

[16] Northam E.A., Anderson P.J., Jacobs R., Hughes M., Warne G.L., Werther G.A. (2001). Neuropsychological profiles of children with type 1 diabetes 6 years after disease onset. Diabetes Care.

[17] Gaudieri P.A., Chen R., Greer T.F., Holmes C.S. (2008). Cognitive function in children with type 1 diabetes: A meta-analysis. Diabetes Care.

[18] Ryan C.M., Geckle M.O., Orchard T.J. (2003). Cognitive efficiency declines over time in adults with Type 1 diabetes: Effects of micro- and macrovascular complications. Diabetologia.

[19] Ott A., Stolk R.P., van Harskamp F., Pols H.A., Hofman A., Breteler M.M. (1999). Diabetes mellitus and the risk of dementia: The Rotterdam Study. Neurology.

[20] Shuba N., Karan (2012). Assessment of the cognitive status in diabetes mellitus. J. Clin. Diagn. Res..

[21] Yau P.L., Javier D.C., Ryan C.M., Tsui W.H., Ardekani B.A., Ten S., Convit A. (2010). Preliminary evidence for brain complications in obese adolescents with type 2 diabetes mellitus. Diabetologia.

[22] Roberts R.O., Knopman D.S., Przybelski S.A., Mielke M.M., Kantarci K., Preboske G.M., Senjem M.L., Pankratz V.S., Geda Y.E., Boeve B.F. (2014). Association of type 2 diabetes with brain atrophy and cognitive impairment. Neurology.

[23] Yeung S.E., Fischer A.L., Dixon R.A. (2009). Exploring effects of type 2 diabetes on cognitive functioning in older adults. Neuropsychology.

[24] Palta P., Schneider A.L., Biessels G.J., Touradji P., Hill-Briggs F. (2014). Magnitude of cognitive dysfunction in adults with type 2 diabetes: A meta-analysis of six cognitive domains and the most frequently reported neuropsychological tests within domains. J. Int. Neuropsychol. Soc..

[25] Ding J., Strachan M.W., Reynolds R.M., Frier B.M., Deary I.J., Fowkes F.G., Lee A.J., McKnight J., Halpin P., Swa K. (2010). Diabetic retinopathy and cognitive decline in older people with type 2 diabetes: The Edinburgh Type 2 Diabetes Study. Diabetes.

[26] Barzilay J.I., Lovato J.F., Murray A.M., Williamson J., Ismail-Beigi F., Karl D., Papademetriou V., Launer L.J. (2013). Albuminuria and cognitive decline in people with diabetes and normal renal function. Clin. J. Am. Soc. Nephrol..

[27] Umegaki H., Hayashi T., Nomura H., Yanagawa M., Nonogaki Z., Nakshima H., Kuzuya M. (2013). Cognitive dysfunction: An emerging concept of a new diabetic complication in the elderly. Geriatr. Gerontol. Int..

[28] Haroon N.N., Austin P.C., Shah B.R., Wu J., Gill S.S., Booth G.L. (2015). Risk of dementia in seniors with newly diagnosed diabetes: A population-based study. Diabetes Care.

[29] Nelson P.T., Smith C.D., Abner E.A., Schmitt F.A., Scheff S.W., Davis G.J., Keller J.N., Jicha G.A., Davis D., Wang-Xia W. (2009). Human cerebral neuropathology of type 2 diabetes mellitus. Biochim. Biophys. Acta.

[30] Liu Y., Liu L., Lu S., Wang D., Liu X., Xie L., Wang G. (2011). Impaired amyloid β-degrading enzymes in brain of streptozotocin-induced diabetic rats. J. Endocrinol. Investig..

[31] Kim B., Backus C., Oh S., Hayes J.M., Feldman E.L. (2009). Increased tau phosphorylation and cleavage in mouse models of type 1 and type 2 diabetes. Endocrinology.

[32] Mimenza-Alvarado A.J., Jiménez-Castillo G.A., Yeverino-Castro S.G., Barragán-Berlanga A.J., Pérez-Zepeda M.U., Ávila-Funes J.A., Aguilar-Navarro S.G. (2020). Effect of poor glycemic control in cognitive performance in the elderly with type 2 diabetes mellitus: The Mexican Health and Aging Study. BMC Geriatr..

[33] Cheng H., Gang X., Liu Y., Wang G., Zhao X., Wang G. (2020). Mitochondrial dysfunction plays a key role in the development of neurodegenerative diseases in diabetes. Am. J. Physiol. Endocrinol. Metab..

[34] Nagatsu T., Nakashima A., Ichinose H., Kobayashi K. (2019). Human tyrosine hydroxylase in Parkinson’s disease and in related disorders. J. Neural Transm..

[35] Silm K., Yang J., Marcott P.F., Asensio C.S., Eriksen J., Guthrie D.A., Newman A.H., Ford C.P., Edwards R.H. (2019). Synaptic Vesicle Recycling Pathway Determines Neurotransmitter Content and Release Properties. Neuron.

[36] Ito H., Kodaka F., Takahashi H., Takano H., Arakawa R., Shimada H., Suhara T. (2011). Relation between Presynaptic and Postsynaptic Dopaminergic Functions Measured by Positron Emission Tomography: Implication of Dopaminergic Tone. J. Neurosci..

[37] Lévesque D., Diaz J., Pilon C., Martres M.P., Giros B., Souil E., Schott D., Morgat J.L., Schwartz J.C., Sokoloff P. (1992). Identification, characterization, and localization of the dopamine D3 receptor in rat brain using 7-[3H]hydroxy-N,N-di-n-propyl-2-aminotetralin. Proc. Natl. Acad. Sci. USA.

[38] Gardner B., Hall D.A., Strange P.G. (1996). Pharmacological analysis of dopamine stimulation of [35S]-GTP gamma S binding via human D2short and D2long dopamine receptors expressed in recombinant cells. Br. J. Pharmacol..

[39] Gardner B., Strange P.G. (1997). Agonist action at D2 (long) dopamine receptors: Ligand binding and functional assays. Br. J. Pharmacol..

[40] Floresco S.B., West A.R., Ash B., Moore H., Grace A.A. (2003). Afferent modulation of dopamine neuron firing differentially regulates tonic and phasic dopamine transmission. Nat. Neurosci..

[41] Fagan R.R., Kearney P.J., Melikian H.E. (2020). In Situ Regulated Dopamine Transporter Trafficking: There’s No Place Like Home. Neurochem. Res..

[42] Kaur S., Singh S., Jaiswal G., Kumar S., Hourani W., Gorain B., Kumar P. (2020). Pharmacology of Dopamine and Its Receptors. Frontiers in Pharmacology of Neurotransmitters.

[43] Pelton E.W., Kimelberg H.K., Shipherd S.V., Bourke R.S. (1981). Dopamine and norepinephrine uptake and metabolism by astroglial cells in culture. Life Sci..

[44] Chen J., Song J., Yuan P., Tian Q., Ji Y., Ren-Patterson R., Liu G., Sei Y., Weinberger D.R. (2011). Orientation and cellular distribution of membrane-bound catechol-O-methyltransferase in cortical neurons: Implications for drug development. J. Biol. Chem..

[45] Klein M.O., Battagello D.S., Cardoso A.R., Hauser D.N., Bittencourt J.C., Correa R.G. (2019). Dopamine: Functions, Signaling, and Association with Neurological Diseases. Cell. Mol. Neurobiol..

[46] Beaulieu J.M., Espinoza S., Gainetdinov R.R. (2015). Dopamine receptors IUPHAR Review 13. Br. J. Pharmacol..

[47] Beaulieu J.M., Gainetdinov R.R. (2011). The Physiology, Signaling, and Pharmacology of Dopamine Receptors. Pharmacol. Rev..

[48] Baik J.H. (2013). Dopamine signaling in reward-related behaviors. Front. Neural Circuits.

[49] Cohen A.I., Todd R.D., Harmon S., O’Malley K.L. (1992). Photoreceptors of mouse retinas possess D4 receptors coupled to adenylate cyclase. Proc. Natl. Acad. Sci. USA.

[50] Kebabian J.W. (1978). Multiple classes of dopamine receptors in mammalian central nervous system: The involvement of dopamine-sensitive adenylyl cyclase. Life Sci..

[51] Cantrell A.R., Smith R.D., Goldin A.L., Scheuer T., Catterall W.A. (1997). Dopaminergic modulation of sodium current in hippocampal neurons via cAMP-dependent phosphorylation of specific sites in the sodium channel alpha subunit. J. Neurosci..

[52] Felder C.C., Jose P.A., Axelrod J. (1989). The dopamine-1 agonist, SKF 82526, stimulates phospholipase-C activity independent of adenylate cyclase. J. Pharmacol. Exp. Ther..

[53] Ugi S., Imamura T., Maegawa H., Egawa K., Yoshizaki T., Shi K., Obata T., Ebina Y., Kashiwagi A., Olefsky J.M. (2004). Protein phosphatase 2A negatively regulates insulin’s metabolic signaling pathway by inhibiting Akt (protein kinase B) activity in 3T3-L1 adipocytes. Mol. Cell. Biol..

[54] Laporte S.A., Miller W.E., Kim K.M., Caron M.G. (2002). beta-Arrestin/AP-2 interaction in G protein-coupled receptor internalization: Identification of a beta-arrestin binging site in beta 2-adaptin. J. Biol. Chem..

[55] Bernheimer H., Hornykiewicz O. (1965). Decreased homovanillic acid concentration in the brain in parkinsonian subjects as an expression of a disorder of central dopamine metabolism. Klin. Wochenschr..

[56] Swerdlow N.R., Koob G.F. (1987). Dopamine, schizophrenia, mania, and depression: Toward a unified hypothesis of cortico-striatopallido-thalamic function. Behav. Brain Sci..

[57] Del Campo N., Chamberlain S.R., Sahakian B.J., Robbins T.W. (2011). The roles of dopamine and noradrenaline in the pathophysiology and treatment of attention-deficit/hyperactivity disorder. Biol. Psychiatry.

[58] Guo J.Y., Ragland J.D., Carter C.S. (2018). Memory and cognition in schizophrenia. Mol. Psychiatry.

[59] Martinussen R., Hayden J., Hogg-Johnson S., Tannock R. (2005). A meta-analysis of working memory impairments in children with attention-deficit/hyperactivity disorder. J. Am. Acad. Child Adolesc. Psychiatry.

[60] Aarsland D., Creese B., Politis M., Chaudhuri K.R., Ffytche D.H., Weintraub D., Ballard C. (2017). Cognitive decline in Parkinson disease. Nat. Rev. Neurol..

[61] Dalrymple-Alford J.C., Kalders A.S., Jones R.D., Watson R.W. (1994). A central executive deficit in patients with Parkinson’s disease. J. Neurol. Neurosurg. Psychiatry.

[62] Downes J.J., Roberts A.C., Sahakian B.J., Evenden J.L., Morris R.G., Robbins T.W. (1989). Impaired extra-dimensional shift performance in medicated and unmedicated Parkinson’s disease: Evidence for a specific attentional dysfunction. Neuropsychologia.

[63] Boller F., Passafiume D., Keefe N.C., Rogers K., Morrow L., Kim Y. (1984). Visuospatial impairment in Parkinson’s disease. Role of perceptual and motor factors. Arch. Neurol..

[64] Warburton J.W. (1967). Memory disturbance and the Parkinson syndrome. Br. J. Med. Psychol..

[65] Wilson R.S., Kaszniak A.W., Klawans H.L., Garron D.C. (1980). High speed memory scanning in parkinsonism. Cortex.

[66] Thabit H., Kyaw Tun T., McDermott J., Sreenan S. (2012). Executive function and diabetes mellitus—A stone left unturned?. Curr. Diabetes Rev..

[67] Vincent C., Hall P.A. (2015). Executive Function in Adults with Type 2 Diabetes: A Meta-Analytic Review. Psychosom. Med..

[68] Liao J.L., Xiong Z.Y., Yang Z.K. (2017). An association of cognitive impairment with diabetes and retinopathy in end stage renal disease patients under peritoneal dialysis. PLoS ONE.

[69] Sadanand S., Balachandar R., Bharath S. (2016). Memory and executive functions in persons with type 2 diabetes: A meta-analysis. Diabetes Metab. Res. Rev..

[70] Brück A., Portin R., Lindell A., Laihinen A., Bergman J., Haaparanta M., Solin O., Rinne J.O. (2001). Positron emission tomography shows that impaired frontal lobe functioning in Parkinson’s disease is related to dopaminergic hypofunction in the caudate nucleus. Neurosci. Lett..

[71] Brück A., Aalto S., Nurmi E., Bergman J., Rinne J.O. (2005). Cortical 6-[18F]fluoro-L-dopa uptake and frontal cognitive functions in early Parkinson’s disease. Neurobiol. Aging.

[72] Sawamoto N., Piccini P., Hotton G., Pavese N., Thielemans K., Brooks D.J. (2008). Cognitive deficits and striato-frontal dopamine release in Parkinson’s disease. Brain.

[73] Jokinen P., Brück A., Aalto S., Forsback S., Parkkola R., Rinne J.O. (2009). Impaired cognitive performance in Parkinson’s disease is related to caudate dopaminergic hypofunction and hippocampal atrophy. Parkinsonism Relat. Disord..

[74] Lebowitz J.J., Khoshbouei H. (2020). Heterogeneity of dopamine release sites in health and degeneration. Neurobiol. Dis..

[75] Seeman P., Bzowej N.H., Guan H.C., Bergeron C., Becker L.E., Reynolds G.P., Bird E.D., Riederer P., Jellinger K., Watanabe S. (1987). Human brain dopamine receptors in children and aging adults. Synapse.

[76] Volkow N.D., Fowler J.S., Wang G.J., Logan J., Schlyer D., MacGregor R., Hitzemann R., Wolf A.P. (1994). Decreased dopamine transporters with age in health human subjects. Ann. Neurol..

[77] Frey K.A., Koeppe R.A., Kilbourn M.R., Vander Borght T.M., Albin R.L., Gilman S., Kuhl D.E. (1996). Presynaptic monoaminergic vesicles in Parkinson’s disease and normal aging. Ann. Neurol..

[78] Ishibashi K., Ishii K., Oda K., Kawasaki K., Mizusawa H., Ishiwata K. (2009). Regional analysis of age-related decline in dopamine transporters and dopamine D2-like receptors in human striatum. Synapse.

[79] Mozley L.H., Gur R.C., Mozley P.D., Gur R.E. (2001). Striatal dopamine transporters and cognitive functioning in healthy men and women. Am. J. Psychiatry.

[80] Erixon-Lindroth N., Farde L., Wahlin T.B., Sovago J., Halldin C., Bäckman L. (2005). The role of the striatal dopamine transporter in cognitive aging. Psychiatry Res..

[81] Bäckman L., Ginovart N., Dixon R.A., Wahlin T.B., Wahlin A., Halldin C., Farde L. (2000). Age-related cognitive deficits mediated by changes in the striatal dopamine system. Am. J. Psychiatry.

[82] Reeves S.J., Grasby P.M., Howard R.J., Bantick R.A., Asselin M.C., Mehta M.A. (2005). A positron emission tomography (PET) investigation of the role of striatal dopamine (D2) receptor availability in spatial cognition. Neuroimage.

[83] Volkow N.D., Gur R.C., Wang G.J., Fowler J.S., Moberg P.J., Ding Y.S., Hitzemann R., Smith G., Logan J. (1998). Association between decline in brain dopamine activity with age and cognitive and motor impairment in healthy individuals. Am. J. Psychiatry.

[84] Fowler C.J., Wiberg A., Oreland L., Marcusson J., Winblad B. (1980). The effect of age on the activity and molecular properties of human brain monoamine oxidase. J. Neural Transm..

[85] Berry A.S., Shah V.D., Baker S.L., Vogel J.W., O’Neil J.P., Janabi M., Schwimmer H.D., Marks S.M., Jagust W.J. (2016). Aging Affects Dopaminergic Neural Mechanisms of Cognitive Flexibility. J. Neurosci..

[86] Juarez E.J., Castrellon J.J., Green M.A., Crawford J.L., Seaman K.L., Smith C.T., Dang L.C., Matuskey D., Morris E.D., Cowan R.L. (2019). Reproducibility of the correlative triad among aging, dopamine receptor availability, and cognition. Psychol. Aging.

[87] Nobili A., Latagliata E.C., Viscomi M.T., Cavallucci V., Cutuli D., Giacovazzo G., Krashia P., Rizzo F.R., Marino R., Federici M. (2017). Dopamine neuronal loss contributes to memory and reward dysfunction in a model of Alzheimer’s disease. Nat. Commun..

[88] Pavăl D. (2017). A Dopamine Hypothesis of Autism Spectrum Disorder. Dev. Neurosci..

[89] Schwab L.C., Garas S.N., Drouin-Ouellet J., Mason S.L., Stott S.R., Barker R.A. (2015). Dopamine and Huntington’s disease. Expert Rev. Neurother..

[90] Phillips A.G., Ahn S., Floresco S.B. (2004). Magnitude of dopamine release in medial prefrontal cortex predicts accuracy of memory on a delayed response task. J. Neurosci..

[91] Watanabe M., Kodama T., Hikosaka K. (1997). Increase of extracellular dopamine in primate prefrontal cortex during a working memory task. J. Neurophysiol..

[92] Yang C.R., Seamans J.K. (1996). Dopamine D1 receptor actions in layers V–VI rat prefrontal cortex neurons in vitro: Modulation of dendritic-somatic signal integration. J. Neurosci..

[93] Simon H., Taghzouti K., Le Moal M. (1986). Deficits in spatial-memory tasks following lesions of septal dopaminergic terminals in the rat. Behav. Brain Res..

[94] Bubser M., Schmidt W.J. (1990). 6-Hydroxydopamine lesion of the rat prefrontal cortex increases locomotor activity, impairs acquisition of delayed alternation tasks, but does not affect uninterrupted tasks in the radial maze. Behav. Brain Res..

[95] Brozoski T.J., Brown R.M., Rosvold H.E., Goldman P.S. (1979). Cognitive deficit caused by regional depletion of dopamine in prefrontal cortex of rhesus monkey. Science.

[96] Mishkin M. (1957). Effects of small frontal lesions on delayed alternation in monkeys. J. Neurophysiol..

[97] Gross C.G., Weiskrantz L. (1962). Evidence for dissociation of impairment on auditory discrimination and delayed response following lateral frontal lesions in monkeys. Exp. Neurol..

[98] Cools R., Froböse M., Aarts E., Hofmans L. (2019). Dopamine and the motivation of cognitive control. Handb. Clin. Neurol..

[99] Arnsten A.F., Cai J.X., Murphy B.L., Goldman-Rakic P.S. (1994). Dopamine D1 receptor mechanisms in the cognitive performance of young adult and aged monkeys. Psychopharmacology.

[100] Sawaguchi T., Goldman-Rakic P.S. (1991). D1 dopamine receptors in prefrontal cortex: Involvement in working memory. Science.

[101] Williams G.V., Goldman-Rakic P.S. (1995). Modulation of memory fields by dopamine D1 receptors in prefrontal cortex. Nature.

[102] Zahrt J., Taylor J.R., Mathew R.G., Arnsten A.F. (1997). Supranormal stimulation of D1 dopamine receptors in the rodent prefrontal cortex impairs spatial working memory performance. J. Neurosci..

[103] Arnsten A.F. (1998). Catecholamine modulation of prefrontal cortical cognitive function. Trends Cogn. Sci..

[104] Luciana M., Depue R.A., Arbisi P., Leon A. (1992). Facilitation of working memory in humans by a d2 dopamine receptor agonist. J. Cogn. Neurosci..

[105] Kimberg D.Y., D’Esposito M. (2003). Cognitive effects of the dopamine receptor agonist pergolide. Neuropsychologia.

[106] Cools R., D’Esposito M. (2011). Inverted-U-shaped dopamine actions on human working memory and cognitive control. Biol. Psychiatry.

[107] Torstenson R., Hartvig P., Långström B., Bastami S., Antoni G., Tedroff J. (1998). Effect of apomorphine infusion on dopamine synthesis rate relates to dopaminergic tone. Neuropharmacology.

[108] Marino R.A., Levy R. (2019). Differential effects of D1 and D2 dopamine agonists on memory, motivation, learning and response time in non-human primates. Eur. J. Neurosci..

[109] Stern Y., Tetrud J.W., Martin W.R., Kutner S.J., Langston J.W. (1990). Cognitive change following MPTP exposure. Neurology.

[110] Da Cunha C., Angelucci M.E., Canteras N.S., Wonnacott S., Takahashi R.N. (2002). The lesion of the rat substantia nigra pars compacta dopaminergic neurons as a model for Parkinson’s disease memory disabilities. Cell. Mol. Neurobiol..

[111] Braga R., Kouzmine I., Canteras N.S., Da Cunha C. (2005). Lesion of the substantia nigra, pars compacta impairs delayed alternation in a Y-maze in rats. Exp. Neurol..

[112] Da Cunha C., Gevaerd M.S., Vital M.A., Miyoshi E., Andreatini R., Silveira R., Takahashi R.N., Canteras N.S. (2001). Memory disruption in rats with nigral lesions induced by MPTP: A model for early Parkinson’s disease amnesia. Behav. Brain Res..

[113] Tadaiesky M.T., Dombrowski P.A., Figueiredo C.P., Cargnin-Ferreira E., Da Cunha C., Takahashi R.N. (2008). Emotional, cognitive and neurochemical alterations in a premotor stage model of Parkinson’s disease. Neuroscience.

[114] Packard M.G., Cahill L., McGaugh J.L. (1994). Amygdala modulation of hippocampal-dependent and caudate nucleus-dependent memory processes. Proc. Natl. Acad. Sci. USA.

[115] Packard M.G., White N.M. (1991). Dissociation of hippocampus and caudate nucleus memory systems by posttraining intracerebral injection of dopamine agonists. Behav. Neurosci..

[116] Schneider J.S., Sun Z.Q., Roeltgen D.P. (1994). Effects of dihydrexidine, a full dopamine D-1 receptor agonist, on delayed response performance in chronic low dose MPTP-treated monkeys. Brain Res..

[117] Chung S.J., Yoo H.S., Oh J.S., Kim J.S., Ye B.S., Sohn Y.H., Lee P.H. (2018). Effect of striatal dopamine depletion on cognition in de novo Parkinson’s disease. Parkinsonism Relat. Disord..

[118] Granon S., Passetti F., Thomas K.L., Dalley J.W., Everitt B.J., Robbins T.W. (2000). Enhanced and impaired attentional performance after infusion of D1 dopaminergic receptor agents into rat prefrontal cortex. J. Neurosci..

[119] Nieoullon A. (2002). Dopamine and the regulation of cognition and attention. Prog. Neurobiol..

[120] Amalric M., Moukhles H., Nieoullon A., Daszuta A. (1995). Complex deficits on reaction time performance following bilateral intrastriatal 6-OHDA infusion in the rat. Eur. J. Neurosci..

[121] Volkow N.D., Wang G.J., Tomasi D., Kollins S.H., Wigal T.L., Newcorn J.H., Telang F.W., Fowler J.S., Logan J., Wong C.T. (2012). Methylphenidate-elicited dopamine increases in ventral striatum are associated with long-term symptom improvement in adults with attention deficit hyperactivity disorder. J. Neurosci..

[122] Turle-Lorenzo N., Maurin B., Puma C., Chezaubernard C., Morain P., Baunez C., Nieoullon A., Amalric M. (2006). The dopamine agonist piribedil with L-DOPA improves attentional dysfunction: Relevance for Parkinson’s disease. J. Pharmacol. Exp. Ther..

[123] Courtière A., Hardouin J., Goujon A., Vidal F., Hasbroucq T. (2003). Selective effects of low-dose dopamine D1 and D2 receptor antagonists on rat information processing. Behav. Pharmacol..

[124] Passetti F., Levita L., Robbins T.W. (2003). Sulpiride alleviates the attentional impairments of rats with medial prefrontal cortex lesions. Behav. Brain Res..

[125] Kempadoo K.A., Mosharov E.V., Choi S.J., Sulzer D., Kandel E.R. (2016). Dopamine release from the locus coeruleus to the dorsal hippocampus promotes spatial learning and memory. Proc. Natl. Acad. Sci. USA.

[126] Holmboe K., Nemoda Z., Fearon R.M., Csibra G., Sasvari-Szekely M., Johnson M.H. (2010). Polymorphisms in dopamine system genes are associated with individual differences in attention in infancy. Dev. Psychol..

[127] Malhotra A.K., Kestler L.J., Mazzanti C., Bates J.A., Goldberg T., Goldman D. (2002). A functional polymorphism in the COMT gene and performance on a test of prefrontal cognition. Am. J. Psychiatry.

[128] Blasi G., Mattay V.S., Bertolino A., Elvevåg B., Callicott J.H., Das S., Kolachana B.S., Egan M.F., Goldberg T.E., Weinberger D.R. (2005). Effect of catechol-O-methyltransferase val158met genotype on attentional control. J. Neurosci..

[129] Zhang Y., Bertolino A., Fazio L., Blasi G., Rampino A., Romano R., Lee M.L., Xiao T., Papp A., Wang D. (2007). Polymorphisms in human dopamine D2 receptor gene affect gene expression, splicing, and neuronal activity during working memory. Proc. Natl. Acad. Sci. USA.

[130] Wiłkość M., Hauser J., Tomaszewska M., Dmitrzak-Weglarz M., Skibińska M., Szczepankiewicz A., Borkowska A. (2010). Influence of dopaminergic and serotoninergic genes on working memory in healthy subjects. Acta Neurobiol. Exp..

[131] Smith C.T., Wallace D.L., Dang L.C., Aarts E., Jagust W.J., D’Esposito M., Boettiger C.A. (2016). Modulation of impulsivity and reward sensitivity in intertemporal choice by striatal and midbrain dopamine synthesis in healthy adults. J. Neurophysiol..

[132] Mehta M.A., Manes F.F., Magnolfi G., Sahakian B.J., Robbins T.W. (2004). Impaired set-shifting and dissociable effects on tests of spatial working memory following the dopamine D2 receptor antagonist sulpiride in human volunteers. Psychopharmacology.

[133] Eagle D.M., Robbins T.W. (2003). Inhibitory control in rats performing a stop-signal reaction-time task: Effects of lesions of the medial striatum and d-amphetamine. Behav. Neurosci..

[134] Eban-Rothschild A., Rothschild G., Giardino W.J., Jones J.R., de Lecea L. (2016). VTA dopaminergic neurons regulate ethologically relevant sleep-wake behaviors. Nat. Neurosci..

[135] Shpakov A.O., Derkach K.V., Berstein L.M. (2015). Brain signaling systems in the Type 2 diabetes and metabolic syndrome: Promising target to treat and prevent these diseases. Future Sci. OA.

[136] de la Monte S.M., Tong M., Nguyen V., Setshedi M., Longato L., Wands J.R. (2010). Ceramide-mediated insulin resistance and impairment of cognitive-motor functions. J. Alzheimers Dis..

[137] Baranov V.G., Propp M.V., Sokoloverova I.M., Savchenko O.N., Onegova R.F. (1980). Dopamine, noradrenaline and serotonin content in various parts of the hypothalamus in alloxan diabetes. Probl. Endokrinol..

[138] Lacković Z., Salković M. (1990). Streptozotocin and alloxan produce alterations in rat brain monoamines independently of pancreatic beta cells destruction. Life Sci..

[139] Lacković Z., Salković M., Kuci Z., Relja M. (1990). Effect of long-lasting diabetes mellitus on rat and human brain monoamines. J. Neurochem..

[140] Ezzeldin E., Souror W.A., El-Nahhas T., Soudi A.N., Shahat A.A. (2014). Biochemical and neurotransmitters changes associated with tramadol in streptozotocin-induced diabetes in rats. BioMed Res. Int..

[141] Rowland N.E., Bellush L.L. (1989). Diabetes mellitus: Stress, neurochemistry and behavior. Neurosci. Biobehav. Rev..

[142] Verberne A.J., Korim W.S., Sabetghadam A., Llewellyn-Smith I.J. (2016). Adrenaline: Insights into its metabolic roles in hypoglycaemia and diabetes. Br. J. Pharmacol..

[143] Babić Leko M., Hof P.R., Šimić G. (2021). Alterations and interactions of subcortical modulatory systems in Alzheimer’s disease. Prog. Brain Res..

[144] Kloiber S., Kohli M.A., Brueckl T., Ripke S., Ising M., Uhr M., Menke A., Unschuld P.G., Horstmann S., Salyakina D. (2010). Variations in tryptophan hydroxylase 2 linked to decreased serotonergic activity are associated with elevated risk for metabolic syndrome in depression. Mol. Psychiatry.

[145] Zhou L., Sutton G.M., Rochford J.J., Semple R.K., Lam D.D., Oksanen L.J., Thornton-Jones Z.D., Clifton P.G., Yueh C.Y., Evans M.L. (2007). Serotonin 2C receptor agonists improve type 2 diabetes via melanocortin-4 receptor signaling pathways. Cell Metab..

[146] Goodnick P.J. (2001). Use of antidepressants in treatment of comorbid diabetes mellitus and depression as well as in diabetic neuropathy. Ann. Clin. Psychiatry.

[147] Chu P.C., Lin M.T., Shian L.R., Leu S.Y. (1986). Alterations in physiologic functions and in brain monoamine content in streptozocin-diabetic rats. Diabetes.

[148] Abraham P.M., Paul J., Paulose C.S. (2010). Down regulation of cerebellar serotonergic receptors in streptozotocin induced diabetic rats: Effect of pyridoxine and Aegle marmelose. Brain Res. Bull..

[149] Sandrini M., Vitale G., Vergoni A.V., Ottani A., Bertolini A. (1997). Streptozotocin-induced diabetes provokes changes in serotonin concentration and on 5-HT1A and 5-HT2 receptors in the rat brain. Life Sci..

[150] Kino M., Yamato T., Aomine M. (2004). Simultaneous measurement of nitric oxide, blood glucose, and monoamines in the hippocampus of diabetic rat: An in vivo microdialysis study. Neurochem. Int..

[151] Yamato T., Misumi Y., Yamasaki S., Kino M., Aomine M. (2004). Diabetes mellitus decreases hippocampal release of neurotransmitters: An in vivo microdialysis study of awake, freely moving rats. Diabetes Nutr. Metab..

[152] Ohtani N., Ohta M., Sugano T. (1997). Microdialysis study of modification of hypothalamic neurotransmitters in streptozotocin-diabetic rats. J. Neurochem..

[153] Fernstrom J.D. (2013). Large neutral amino acids: Dietary effects on brain neurochemistry and function. Amino Acids..

[154] Zemdegs J., Quesseveur G., Jarriault D., Pénicaud L., Fioramonti X., Guiard B.P. (2016). High-fat diet-induced metabolic disorders impairs 5-HT function and anxiety-like behavior in mice. Br. J. Pharmacol..

[155] Jorgensen K.D. (1977). Actions of fenfluramine on glucose uptake in vitro and in vivo. Acta Pharmacol. Toxicol..

[156] Russell J.C., Dolphin P.J., Graham S.E., Amy R.M., Brindley D.N. (1998). Improvement of insulin sensitivity and cardiovascular outcomes in the JCR:LA-cp rat by D-fenfluramine. Diabetologia.

[157] Arora R., Dryden S., McKibbin P.E., Williams G. (1994). Acute dexfenfluramine administration normalizes glucose tolerance in rats with insulin-deficient diabetes. Eur. J. Clin. Investig..

[158] Derkach K.V., Bondareva V.M., Chistyakova O.V., Berstein L.M., Shpakov A.O. (2015). The Effect of Long-Term Intranasal Serotonin Treatment on Metabolic Parameters and Hormonal Signaling in Rats with High-Fat Diet/Low-Dose Streptozotocin-Induced Type 2 Diabetes. Int. J. Endocrinol..

[159] Biskup C.S., Sánchez C.L., Arrant A., Van Swearingen A.E., Kuhn C., Zepf F.D. (2012). Effects of acute tryptophan depletion on brain serotonin function and concentrations of dopamine and norepinephrine in C57BL/6J and BALB/cJ mice. PLoS ONE.

[160] Jans L.A., Korte-Bouws G.A., Korte S.M., Blokland A. (2010). The effects of acute tryptophan depletion on affective behaviour and cognition in Brown Norway and Sprague Dawley rats. J. Psychopharmacol..

[161] Young S.N., Ervin F.R., Pihl R.O., Finn P. (1989). Biochemical aspects of tryptophan depletion in primates. Psychopharmacology.

[162] Young S.N., Smith S.E., Pihl R.O., Ervin F.R. (1985). Tryptophan depletion causes a rapid lowering of mood in normal males. Psychopharmacology.

[163] Riedel W.J., Klaassen T., Deutz N.E., van Someren A., van Praag H.M. (1999). Tryptophan depletion in normal volunteers produces selective impairment in memory consolidation. Psychopharmacology.

[164] Rodríguez J.J., Noristani H.N., Verkhratsky A. (2012). The serotonergic system in ageing and Alzheimer’s disease. Prog. Neurobiol..

[165] Whiley L., Chappell K.E., D’Hondt E., Lewis M.R., Jiménez B., Snowden S.G., Soininen H., Kłoszewska I., Mecocci P., Tsolaki M. (2021). Metabolic phenotyping reveals a reduction in the bioavailability of serotonin and kynurenine pathway metabolites in both the urine and serum of individuals living with Alzheimer’s disease. Alzheimers Res. Ther..

[166] Jenkins T.A., Nguyen J.C., Polglaze K.E., Bertrand P.P. (2016). Influence of Tryptophan and Serotonin on Mood and Cognition with a Possible Role of the Gut-Brain Axis. Nutrients.

[167] Sukhov I.B., Chistyakova O.V., Shipilov V.N., Doilnitsyn A.M., Shpakov A.O. (2015). Spatial memory and regulation of brain adenylyl cyclase by serotonin and dopamine in rat with streptozotocin diabetes. Ross. Fiziol. Zhurnal Im. IM Sechenova.

[168] Pan Y., Gembom E., Peng W., Lesch K.P., Mossner R., Simantov R. (2001). Plasticity in serotonin uptake in primary neuronal cultures of serotonin transporter knockout mice. Brain Res. Dev. Brain Res..

[169] Gardier A.M., Moratalla R., Cuéllar B., Sacerdote M., Guibert B., Lebrec H., Graybiel A.M. (2000). Interaction between the serotoninergic and dopaminergic systems in d-fenfluramine-induced activation of c-fos and jun B genes in rat striatal neurons. J. Neurochem..

[170] Rouillard C., Bovetto S., Gervais J., Richard D. (1996). Fenfluramine-induced activation of the immediate-early gene c-fos in the striatum: Possible interaction between serotonin and dopamine. Brain Res. Mol. Brain Res..

[171] Yadid G., Pacak K., Kopin I.J., Goldstein D.S. (1994). Endogenous serotonin stimulates striatal dopamine release in conscious rats. J. Pharmacol. Exp. Ther..

[172] Doherty M.D., Pickel V.M. (2000). Ultrastructural localization of the serotonin 2A receptor in dopaminergic neurons in the ventral tegmental area. Brain Res..

[173] Nocjar C., Roth B.L., Pehek E.A. (2002). Localization of 5-HT(2A) receptors on dopamine cells in subnuclei of the midbrain A10 cell group. Neuroscience.

[174] de Bartolomeis A., Fiore G. (2004). Postsynaptic density scaffolding proteins at excitatory synapse and disorders of synaptic plasticity: Implications for human behavior pathologies. Int. Rev. Neurobiol..

[175] de Bartolomeis A., Buonaguro E.F., Iasevoli F. (2013). Serotonin-glutamate and serotonin-dopamine reciprocal interactions as putative molecular targets for novel antipsychotic treatments: From receptor heterodimers to postsynaptic scaffolding and effector proteins. Psychopharmacology.

[176] Ter Horst K.W., Lammers N.M., Trinko R., Opland D.M., Figee M., Ackermans M.T., Booij J., van den Munckhof P., Schuurman P.R., Fliers E. (2018). Striatal dopamine regulates systemic glucose metabolism in humans and mice. Sci. Transl. Med..

[177] Wang G.J., Volkow N.D., Logan J., Pappas N.R., Wong C.T., Zhu W., Netusil N., Fowler J.S. (2001). Brain dopamine and obesity. Lancet.

[178] de Weijer B.A., van de Giessen E., van Amelsvoort T.A., Boot E., Braak B., Janssen I.M., van de Laar A., Fliers E., Serlie M.J., Booij J. (2011). Lower striatal dopamine D2/3 receptor availability in obese compared with non-obese subjects. EJNMMI Res..

[179] García-Tornadú I., Ornstein A.M., Chamson-Reig A., Wheeler M.B., Hill D.J., Arany E., Rubinstein M., Becu-Villalobos D. (2010). Disruption of the dopamine d2 receptor impairs insulin secretion and causes glucose intolerance. Endocrinology.

[180] Lacau-Mengido I.M., Mejía M.E., Díaz-Torga G.S., Gonzalez Iglesias A., Formía N., Libertun C., Becú-Villalobos D. (2000). Endocrine studies in ivermectin-treated heifers from birth to puberty. J. Anim. Sci..

[181] Díaz-Torga G.S., Mejia M.E., González-Iglesias A., Formia N., Becú-Villalobos D., Lacau-Mengido I.M. (2001). Metabolic cues for puberty onset in free grazing Holstein heifers naturally infected with nematodes. Theriogenology.

[182] Joseph J.W., Koshkin V., Saleh M.C., Sivitz W.I., Zhang C.Y., Lowell B.B., Chan C.B., Wheeler M.B. (2004). Free fatty acid-induced beta-cell defects are dependent on uncoupling protein 2 expression. J. Biol. Chem..

[183] Tarricone I., Casoria M., Gozzi B.F., Grieco D., Menchetti M., Serretti A., Ujkaj M., Pastorelli F., Berardi D. (2006). Metabolic risk factor profile associated with use of second generation antipsychotics: A cross sectional study in a Community Mental Health Centre. BMC Psychiatry.

[184] Ballon J.S., Pajvani U., Freyberg Z., Leibel R.L., Lieberman J.A. (2014). Molecular pathophysiology of metabolic effects of antipsychotic medications. Trends Endocrinol. Metab..

[185] Anitha M., Abraham P.M., Paulose C.S. (2012). Striatal dopamine receptors modulate the expression of insulin receptor, IGF-1 and GLUT-3 in diabetic rats: Effect of pyridoxine treatment. Eur. J. Pharmacol..

[186] Pérez-Taboada I., Alberquilla S., Martín E.D., Anand R., Vietti-Michelina S., Tebeka N.N., Cantley J., Cragg S.J., Moratalla R., Vallejo M. (2020). Diabetes Causes Dysfunctional Dopamine Neurotransmission Favoring Nigrostriatal Degeneration in Mice. Mov. Disord..

[187] Mielke J.G., Wang Y.T. (2005). Insulin exerts neuroprotection by counteracting the decrease in cell-surface GABA receptors following oxygen-glucose deprivation in cultured cortical neurons. J. Neurochem..

[188] Ramalingam M., Kim S.J. (2014). The role of insulin against hydrogen peroxide-induced oxidative damages in differentiated SH-SY5Y cells. J. Recept. Signal Transduct..

[189] Pang Y., Lin S., Wright C., Shen J., Carter K., Bhatt A., Fan L.W. (2016). Intranasal insulin protects against substantia nigra dopaminergic neuronal loss and alleviates motor deficits induced by 6-OHDA in rats. Neuroscience.

[190] Carvelli L., Morón J.A., Kahlig K.M., Ferrer J.V., Sen N., Lechleiter J.D., Leeb-Lundberg L.M., Merrill G., Lafer E.M., Ballou L.M. (2002). PI 3-kinase regulation of dopamine uptake. J. Neurochem..

[191] Garcia B.G., Wei Y., Moron J.A., Lin R.Z., Javitch J.A., Galli A. (2005). Akt is essential for insulin modulation of amphetamine-induced human dopamine transporter cell-surface redistribution. Mol. Pharmacol..

[192] Owens W.A., Sevak R.J., Galici R., Chang X., Javors M.A., Galli A., France C.P., Daws L.C. (2005). Deficits in dopamine clearance and locomotion in hypoinsulinemic rats unmask novel modulation of dopamine transporters by amphetamine. J. Neurochem..

[193] Wei Y., Williams J.M., Dipace C., Sung U., Javitch J.A., Galli A., Saunders C. (2007). Dopamine transporter activity mediates amphetamine-induced inhibition of Akt through a Ca2+/calmodulin-dependent kinase II-dependent mechanism. Mol. Pharmacol..

[194] Könner A.C., Hess S., Tovar S., Mesaros A., Sánchez-Lasheras C., Evers N., Verhagen L.A., Brönneke H.S., Kleinridders A., Hampel B. (2011). Role for insulin signaling in catecholaminergic neurons in control of energy homeostasis. Cell Metab..

[195] Hong C.T., Chen K.Y., Wang W., Chiu J.Y., Wu D., Chao T.Y., Hu C.J., Chau K.D., Bamodu O.A. (2020). Insulin Resistance Promotes Parkinson’s Disease through Aberrant Expression of α-Synuclein, Mitochondrial Dysfunction, and Deregulation of the Polo-Like Kinase 2 Signaling. Cells.

[196] Kleinridders A., Cai W., Cappellucci L., Ghazarian A., Collins W.R., Vienberg S.G., Pothos E.N., Kahn C.R. (2015). Insulin resistance in brain alters dopamine turnover and causes behavioral disorders. Proc. Natl. Acad. Sci. USA.

[197] Potter G.M., Moshirfar A., Castonguay T.W. (1999). Insulin affects dopamine overflow in the nucleus accumbens and the striatum. Physiol. Behav..

[198] Fiory F., Mirra P., Nigro C., Pignalosa F.C., Zatterale F., Ulianich L., Prevete N., Formisano P., Beguinot F., Miele C. (2018). Role of the HIF-1α/Nur77 axis in the regulation of the tyrosine hydroxylase expression by insulin in PC12 cells. J. Cell. Physiol..

[199] Patterson T.A., Brot M.D., Zavosh A., Schenk J.O., Szot P., Figlewicz D.P. (1998). Food deprivation decreases mRNA and activity of the rat dopamine transporter. Neuroendocrinology.

[200] Speed N.K., Matthies H.J., Kennedy J.P., Vaughan R.A., Javitch J.A., Russo S.J., Lindsley C.W., Niswender K., Galli A. (2010). Akt-dependent and isoform-specific regulation of dopamine transporter cell surface expression. ACS Chem. Neurosci..

[201] Bournival J., Francoeur M.A., Renaud J., Martinoli M.G. (2012). Quercetin and sesamin protect neuronal PC12 cells from high-glucose-induced oxidation, nitrosative stress, and apoptosis. Rejuvenation Res..

[202] Renaud J., Bournival J., Zottig X., Martinoli M.G. (2014). Resveratrol protects DAergic PC12 cells from high glucose-induced oxidative stress and apoptosis: Effect on p53 and GRP75 localization. Neurotox Res..

[203] Su C.J., Shen Z., Cui R.X., Huang Y., Xu D.L., Zhao F.L., Pan J., Shi A.M., Liu T., Yu Y.L. (2020). Thioredoxin-Interacting Protein (TXNIP) Regulates Parkin/PINK1-mediated Mitophagy in Dopaminergic Neurons Under High-glucose Conditions: Implications for Molecular Links Between Parkinson’s Disease and Diabetes. Neurosci. Bull..

[204] Koshimura K., Tanaka J., Murakami Y., Kato Y. (2003). Effect of high concentration of glucose on dopamine release from pheochromocytoma-12 cells. Metabolism.

[205] Umhau J.C., Petrulis S.G., Diaz R., Rawlings R., George D.T. (2003). Blood glucose is correlated with cerebrospinal fluid neurotransmitter metabolites. Neuroendocrinology.

[206] Levin B.E. (2000). Glucose-regulated dopamine release from substantia nigra neurons. Brain Res..

[207] Pinkas A., Lawes M., Aschner M. (2018). System-specific neurodegeneration following glucotoxicity in the *C. elegans* model. Neurotoxicology.

[208] Ramakrishnan R., Kempuraj D., Prabhakaran K., Jayakumar A.R., Devi R.S., Suthanthirarajan N., Namasivayam A. (2005). A short-term diabetes induced changes of catecholamines and p38-MAPK in discrete areas of rat brain. Life Sci..

[209] Robinson R., Krishnakumar A., Paulose C.S. (2009). Enhanced dopamine D1 and D2 receptor gene expression in the hippocampus of hypoglycaemic and diabetic rats. Cell. Mol. Neurobiol..

[210] Gallego M., Setién R., Izquierdo M.J., Casis O., Casis E. (2003). Diabetes-induced biochemical changes in central and peripheral catecholaminergic systems. Physiol. Res..

[211] Renaud J., Bassareo V., Beaulieu J., Pinna A., Schlich M., Lavoie C., Murtas D., Simola N., Martinoli M.G. (2018). Dopaminergic neurodegeneration in a rat model of long-term hyperglycemia: Preferential degeneration of the nigrostriatal motor pathway. Neurobiol. Aging.

[212] Kwok R.P., Juorio A.V. (1986). Concentration of striatal tyramine and dopamine metabolism in diabetic rats and effect of insulin administration. Neuroendocrinology.

[213] Bitar M., Koulu M., Rapoport S.I., Linnoila M. (1986). Diabetes-induced alteration in brain monoamine metabolism in rats. J. Pharmacol. Exp. Ther..

[214] Figlewicz D.P., Brot M.D., McCall A.L., Szot P. (1996). Diabetes causes differential changes in CNS noradrenergic and dopaminergic neurons in the rat: A molecular study. Brain Res..

[215] Glanville N.T., Anderson G.H. (1986). Hypothalamic catecholamine metabolism in diabetic rats: The effect of insulin deficiency and meal ingestion. J. Neurochem..

[216] Nascimento P.S., Lovatel G.A., Barbosa S., Ilha J., Centenaro L.A., Malysz T., Xavier L.L., Schaan B.D., Achaval M. (2011). Treadmill training improves motor skills and increases tyrosine hydroxylase immunoreactivity in the substantia nigra pars compacta in diabetic rats. Brain Res..

[217] Kono T., Takada M. (1994). Dopamine depletion in nigrostriatal neurons in the genetically diabetic rat. Brain Res..

[218] Azevedo M., Fernandes F., Lisboa P., Manso C. (1980). Platelet monoamine oxidase activity in diabetics. Acta Med. Port..

[219] Xiu L., Lin M., Liu W., Kong D., Liu Z., Zhang Y., Ouyang P., Liang Y., Zhong S., Chen C. (2015). Association of DRD3, COMT, and SLC6A4 Gene Polymorphisms with Type 2 Diabetes in Southern Chinese: A Hospital-Based Case-Control Study. Diabetes Technol. Ther..

[220] Kring S.I., Werge T., Holst C., Toubro S., Astrup A., Hansen T., Pedersen O., Sørensen T.I. (2009). Polymorphisms of serotonin receptor 2A and 2C genes and COMT in relation to obesity and type 2 diabetes. PLoS ONE.

[221] Xiu L., Liu W., Zhou S., Lin M., Ouyang P., Qin J., Zhao X., Kong D., Rao S., Ding Y. (2014). Study on the association between catechol-O-methyltransferase gene polymorphisms and type 2 diabetes mellitus. Zhonghua Liu Xing Bing Xue Za Zhi.

[222] Pagano G., Polychronis S., Wilson H., Giordano B., Ferrara N., Niccolini F., Politis M. (2018). Diabetes mellitus and Parkinson disease. Neurology.

[223] Brownlee M. (2005). The pathobiology of diabetic complications: A unifying mechanism. Diabetes.

[224] Zheng H., Wu J., Jin Z., Yan L.J. (2016). Protein Modifications as Manifestations of Hyperglycemic Glucotoxicity in Diabetes and Its Complications. Biochem. Insights.

[225] Nigro C., Leone A., Raciti G.A., Longo M., Mirra P., Formisano P., Beguinot F., Miele C. (2017). Methylglyoxal-Glyoxalase 1 Balance: The Root of Vascular Damage. Int. J. Mol. Sci..

[226] van Sloten T.T. (2017). Vascular dysfunction: At the heart of cardiovascular disease, cognitive impairment and depressive symptoms. Artery Res..

[227] Nigro C., Leone A., Fiory F., Prevenzano I., Nicolò A., Mirra P., Beguinot F., Miele C. (2019). Dicarbonyl Stress at the Crossroads of Healthy and Unhealthy Aging. Cells.

[228] Fiory F., Lombardi A., Miele C., Giudicelli J., Beguinot F., Van Obberghen E. (2011). Methylglyoxal impairs insulin signalling and insulin action on glucose-induced insulin secretion in the pancreatic beta cell line INS-1E. Diabetologia.

[229] Riboulet-Chavey A., Pierron A., Durand I., Murdaca J., Giudicelli J., Van Obberghen E. (2006). Methylglyoxal impairs the insulin signaling pathways independently of the formation of intracellular reactive oxygen species. Diabetes.

[230] Nigro C., Raciti G.A., Leone A., Fleming T.H., Longo M., Prevenzano I., Fiory F., Mirra P., D’Esposito V., Ulianich L. (2014). Methylglyoxal impairs endothelial insulin sensitivity both in vitro and in vivo. Diabetologia.

[231] Mirra P., Nigro C., Prevenzano I., Procopio T., Leone A., Raciti G.A., Andreozzi F., Longo M., Fiory F., Beguinot F. (2017). The role of miR-190a in methylglyoxal-induced insulin resistance in endothelial cells. Biochim. Biophys. Acta Mol. Basis Dis..

[232] Nigro C., Mirra P., Prevenzano I., Leone A., Fiory F., Longo M., Cabaro S., Oriente F., Beguinot F., Miele C. (2018). miR-214-Dependent Increase of PHLPP2 Levels Mediates the Impairment of Insulin-Stimulated Akt Activation in Mouse Aortic Endothelial Cells Exposed to Methylglyoxal. Int. J. Mol. Sci..

[233] Nigro C., Leone A., Longo M., Prevenzano I., Fleming T.H., Nicolò A., Parrillo L., Spinelli R., Formisano P., Nawroth P.P. (2019). Methylglyoxal accumulation de-regulates HoxA5 expression, thereby impairing angiogenesis in glyoxalase 1 knock-down mouse aortic endothelial cells. Biochim. Biophys. Acta Mol. Basis Dis..

[234] Kikuchi S., Shinpo K., Moriwaka F., Makita Z., Miyata T., Tashiro K. (1999). Neurotoxicity of methylglyoxal and 3-deoxyglucosone on cultured cortical neurons: Synergism between glycation and oxidative stress, possibly involved in neurodegenerative diseases. J. Neurosci. Res..

[235] Beeri M.S., Moshier E., Schmeidler J., Godbold J., Uribarri J., Reddy S., Sano M., Grossman H.T., Cai W., Vlassara H. (2011). Serum concentration of an inflammatory glycotoxin, methylglyoxal, is associated with increased cognitive decline in elderly individuals. Mech. Ageing Dev..

[236] Yaffe K., Lindquist K., Schwartz A.V., Vitartas C., Vittinghoff E., Satterfield S., Simonsick E.M., Launer L., Rosano C., Cauley J.A. (2011). Advanced glycation end product level, diabetes, and accelerated cognitive aging. Neurology.

[237] Di Loreto S., Zimmitti V., Sebastiani P., Cervelli C., Falone S., Amicarelli F. (2008). Methylglyoxal causes strong weakening of detoxifying capacity and apoptotic cell death in rat hippocampal neurons. Int. J. Biochem. Cell Biol..

[238] Heimfarth L., Loureiro S.O., Pierozan P., de Lima B.O., Reis K.P., Torres E.B., Pessoa-Pureur R. (2013). Methylglyoxal-induced cytotoxicity in neonatal rat brain: A role for oxidative stress and MAP kinases. Metab. Brain Dis..

[239] Huang X., Wang F., Chen W., Chen Y., Wang N., von Maltzan K. (2012). Possible link between the cognitive dysfunction associated with diabetes mellitus and the neurotoxicity of methylglyoxal. Brain Res..

[240] Hansen F., Pandolfo P., Galland F., Torres F.V., Dutra M.F., Batassini C., Guerra M.C., Leite M.C., Gonçalves C.A. (2016). Methylglyoxal can mediate behavioral and neurochemical alterations in rat brain. Physiol. Behav..

[241] Lissner L.J., Rodrigues L., Wartchow K.M., Borba E., Bobermin L.D., Fontella F.U., Hansen F., Quincozes-Santos A., Souza D.O.G., Gonçalves C.A. (2021). Short-Term Alterations in Behavior and Astroglial Function after Intracerebroventricular Infusion of Methylglyoxal in Rats. Neurochem. Res..

[242] Akhter F., Chen D., Akhter A., Sosunov A.A., Chen A., McKhann G.M., Yan S.F., Yan S.S. (2020). High Dietary Advanced Glycation End Products Impair Mitochondrial and Cognitive Function. J. Alzheimers Dis..

[243] Smith M.A., Rudnicka-Nawrot M., Richey P.L., Praprotnik D., Mulvihill P., Miller C.A., Sayre L.M., Perry G. (1995). Carbonyl-related posttranslational modification of neurofilament protein in the neurofibrillary pathology of Alzheimer’s disease. J. Neurochem..

[244] Vicente Miranda H., El-Agnaf O.M., Outeiro T.F. (2016). Glycation in Parkinson’s disease and Alzheimer’s disease. Mov. Disord..

[245] Haddad M., Perrotte M., Khedher M.R.B., Demongin C., Lepage A., Fülöp T., Ramassamy C. (2019). Methylglyoxal and Glyoxal as Potential Peripheral Markers for MCI Diagnosis and Their Effects on the Expression of Neurotrophic, Inflammatory and Neurodegenerative Factors in Neurons and in Neuronal Derived-Extracellular Vesicles. Int. J. Mol. Sci..

[246] West R.K., Moshier E., Lubitz I., Schmeidler J., Godbold J., Cai W., Uribarri J., Vlassara H., Silverman J.M., Beeri M.S. (2014). Dietary advanced glycation end products are associated with decline in memory in young elderly. Mech. Ageing Dev..

[247] Srikanth V., Westcott B., Forbes J., Phan T.G., Beare R., Venn A., Pearson S., Greenaway T., Parameswaran V., Münch G. (2013). Methylglyoxal, cognitive function and cerebral atrophy in older people. J. Gerontol. A Biol. Sci. Med. Sci..

[248] Song D.W., Xin N., Xie B.J., Li Y.J., Meng L.Y., Li H.M., Schläppi M., Deng Y.L. (2014). Formation of a salsolinol-like compound, the neurotoxin, 1-acetyl-6,7-dihydroxy-1,2,3,4-tetrahydroisoquinoline, in a cellular model of hyperglycemia and a rat model of diabetes. Int. J. Mol. Med..

[249] Deng Y., Zhang Y., Li Y., Xiao S., Song D., Qing H., Li Q., Rajput A.H. (2012). Occurrence and distribution of salsolinol-like compound, 1-acetyl-6,7-dihydroxy-1,2,3,4-tetrahydroisoquinoline (ADTIQ) in parkinsonian brains. J. Neural Transm..

[250] Xie B., Lin F., Ullah K., Peng L., Ding W., Dai R., Qing H., Deng Y. (2015). A newly discovered neurotoxin ADTIQ associated with hyperglycemia and Parkinson’s disease. Biochem. Biophys. Res. Commun..

[251] Farrer M., Kachergus J., Forno L., Lincoln S., Wang D.S., Hulihan M., Maraganore D., Gwinn-Hardy K., Wszolek Z., Dickson D. (2004). Comparison of kindreds with parkinsonism and alpha-synuclein genomic multiplications. Ann. Neurol..

[252] Sharma N., Rao S.P., Kalivendi S.V. (2019). The deglycase activity of DJ-1 mitigates α-synuclein glycation and aggregation in dopaminergic cells: Role of oxidative stress mediated downregulation of DJ-1 in Parkinson’s disease. Free Radic. Biol. Med..

[253] de Almeida G.R.L., Szczepanik J.C., Selhorst I., Schmitz A.E., Dos Santos B., Cunha M.P., Heinrich I.A., de Paula G.C., De Bem A.F., Leal R.B. (2021). Methylglyoxal-Mediated Dopamine Depletion, Working Memory Deficit, and Depression-Like Behavior Are Prevented by a Dopamine/Noradrenaline Reuptake Inhibitor. Mol. Neurobiol..

[254] Szczepanik J.C., de Almeida G.R.L., Cunha M.P., Dafre A.L. (2020). Repeated Methylglyoxal Treatment Depletes Dopamine in the Prefrontal Cortex, and Causes Memory Impairment and Depressive-Like Behavior in Mice. Neurochem. Res..

[255] Xie B., Lin F., Peng L., Ullah K., Wu H., Qing H., Deng Y. (2014). Methylglyoxal increases dopamine level and leads to oxidative stress in SH-SY5Y cells. Acta Biochim. Biophys. Sin..

[256] Simpson I.A., Carruthers A., Vannucci S.J. (2007). Supply and demand in cerebral energy metabolism: The role of nutrient transporters. J. Cereb. Blood Flow Metab..

[257] Sergi D., Renaud J., Simola N., Martinoli M.G. (2019). Diabetes, a Contemporary Risk for Parkinson’s Disease: Epidemiological and Cellular Evidences. Front. Aging Neurosci..

